# Picoinjection Enables Digital Detection of RNA with Droplet RT-PCR

**DOI:** 10.1371/journal.pone.0062961

**Published:** 2013-04-26

**Authors:** Dennis J. Eastburn, Adam Sciambi, Adam R. Abate

**Affiliations:** Department of Bioengineering and Therapeutic Sciences, California Institute for Quantitative Biosciences, University of California San Francisco, San Francisco, California, United States of America; University of California, Merced, United States of America

## Abstract

The ability to add reagents to drops in a sequential fashion is necessary for numerous applications of microfluidics in biology. An important method for accomplishing this is picoinjection, a technique in which reagents are injected into aqueous drops using an electric field. While picoinjection has been shown to allow the precise addition of reagents to drops, its compatibility with biological reactions is yet to be thoroughly demonstrated. Here, we investigate the compatibility of picoinjection with digital RT-PCR Taqman assays, reactions that incorporate nucleic acids, enzymes, and other common biological reagents. We find that picoinjection is compatible with this assay and enables the detection of RNA transcripts at rates comparable to workflows not incorporating picoinjection. We also find that picoinjection results in negligible transfer of material between drops and that the drops faithfully retain their compartmentalization.

## Introduction

Droplet-based microfluidic techniques are continuing to expand into the molecular biology laboratory, due to their versatility and the throughput with which they can analyze heterogeneous samples of nucleic acids, enzymes, and cells [1]–[4]. Microdroplets, tiny spheres of aqueous liquid ranging from 1 to 100 µm in diameter, are used to encapsulate biological components in an oil-based emulsion [5], [6]. The drops serve, essentially, as very tiny “test tubes,” compartmentalizing millions of reactions in only a few hundred microliters of emulsion. A major advantage of droplet-based microfluidics is that it combines very small reagent usage per reaction (∼10^−12^ liters per drop) with ultrahigh-throughput reaction processing (>1,000/s), enabling millions of picoliter volume reactions to be analyzed in a matter of minutes [3].

Most biological assays require the sequential addition of reagents at different times. For microfluidic techniques to be most widely useful, a robust procedure for adding reagents to drops is therefore essential. One technique for accomplishing this is electrocoalescence of drops, in which the reagent is added by merging the drop with a drop of the reagent using an electric field [7]–[9]. Another technique is picoinjection, which injects the reagent directly into the drops by flowing them past a pressurized channel and applying an electric field [10], [11]. An advantage of picoinjection is that it does not require the synchronization of two streams of drops, making it easier to implement and more robust in operation. However, as of yet, the compatibility of picoinjection with biological assays has not been thoroughly demonstrated. In particular, variability in the volume injected from drop to drop and the potential degradation of reagents by the electric field may interfere with assays. In addition, during picoinjection, the drops temporarily merge with the reagent fluid, potentially allowing transfer of material between drops, and cross-contamination. For picoinjection to be validated as a robust and dependable means of adding reagents to drops for biological assays, its impact on biological reactions and the potential for cross-contamination must be characterized.

In this paper, we characterize the impact of picoinjection on biological assays performed in drops and the extent of material transfer between drops. Using sensitive digital RT-PCR assays, we show that picoinjection is a robust method for adding reagents to drops, allowing the detection of RNA transcripts at rates comparable to reactions not incorporating picoinjection. We also find that there is negligible transfer of material between drops. The benefit of workflows incorporating picoinjection over those that do not is that picoinjection allows reagents to be added in a sequential fashion, opening up new possibilities for applying digital RT-PCR to the analysis of heterogeneous populations of nucleic acids, viruses, and cells.

## Results and Discussion

### Detection of RNA transcripts in picoinjected drops

**Figure 1:**
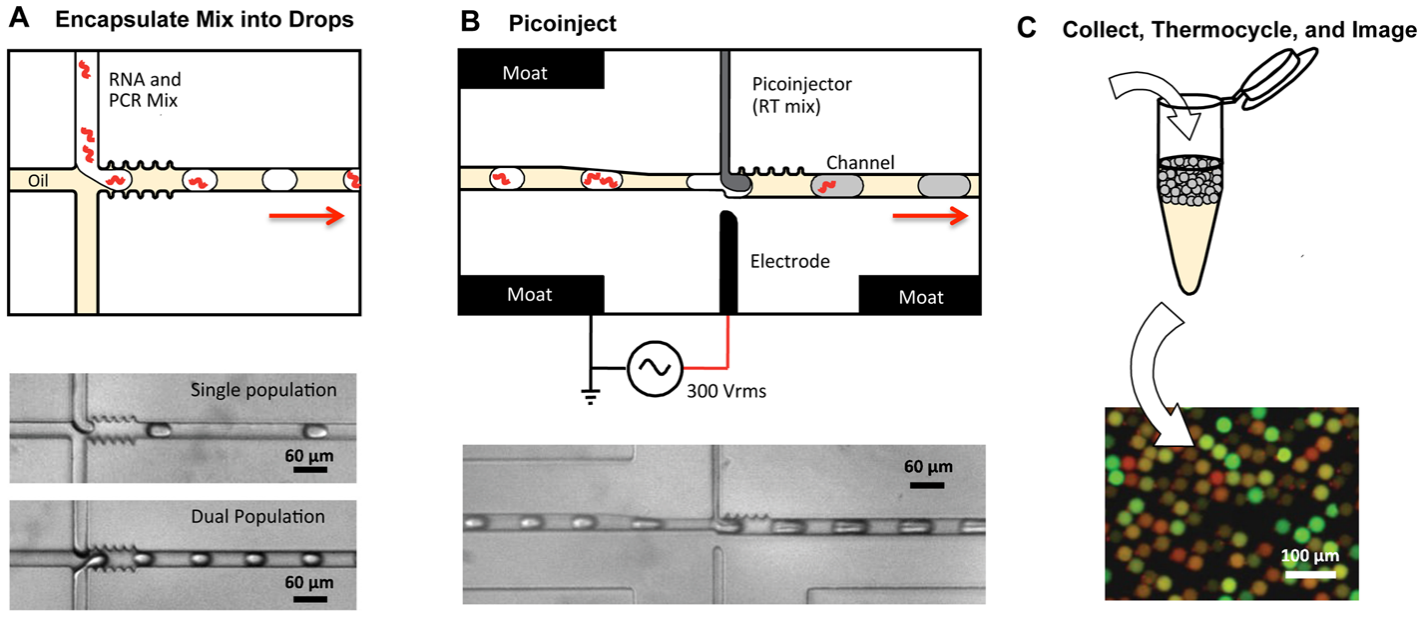
Microfluidic devices and digital RT-PCR workflow used in this study. (A) Drops containing RNA and RT-PCR reagents are created with a microfluidic T-junction and carrier oil. Brightfield microscopy images of the drop formation are shown below, the middle image showing the generation of one population of drops from a single reaction mixture, and the lower the generation of two populations from two mixtures. The red arrows indicate the direction of emulsion flow in the illustrations. (B) After formation, the drops are picoinjected with reverse transcriptase using a picoinjection channel triggered by an electric field, applied by an electrode channel immediately opposite the picoinjector. Picoinjection fluid is pictured as dark gray in the schematic diagram. (C) The picoinjected drops are collected into a tube, thermocycled, and imaged with a fluorescent microscope.

A potential concern of using picoinjection for RT-PCR assays is that it may interfere with reactions in the drops; for example, the process may result in variability in the amount of reagents between the drops or degrade key components upon exposure to the electric field. To investigate these issues, we compared the detection of two cancer-relevant human transcripts, EpCAM and CD44, in picoinjected and non-picoinjected drops using Taqman RT-PCR, (Fig. 1). The Taqman probe for detecting EpCAM was conjugated to the fluorophore 6 carboxyfuoroscein (FAM) and the probe for CD44 to the dye Cy5. The probe mix also contained primers that flank the Taqman probes and yield ∼150 base amplicons from these genes.

**Figure 2:**
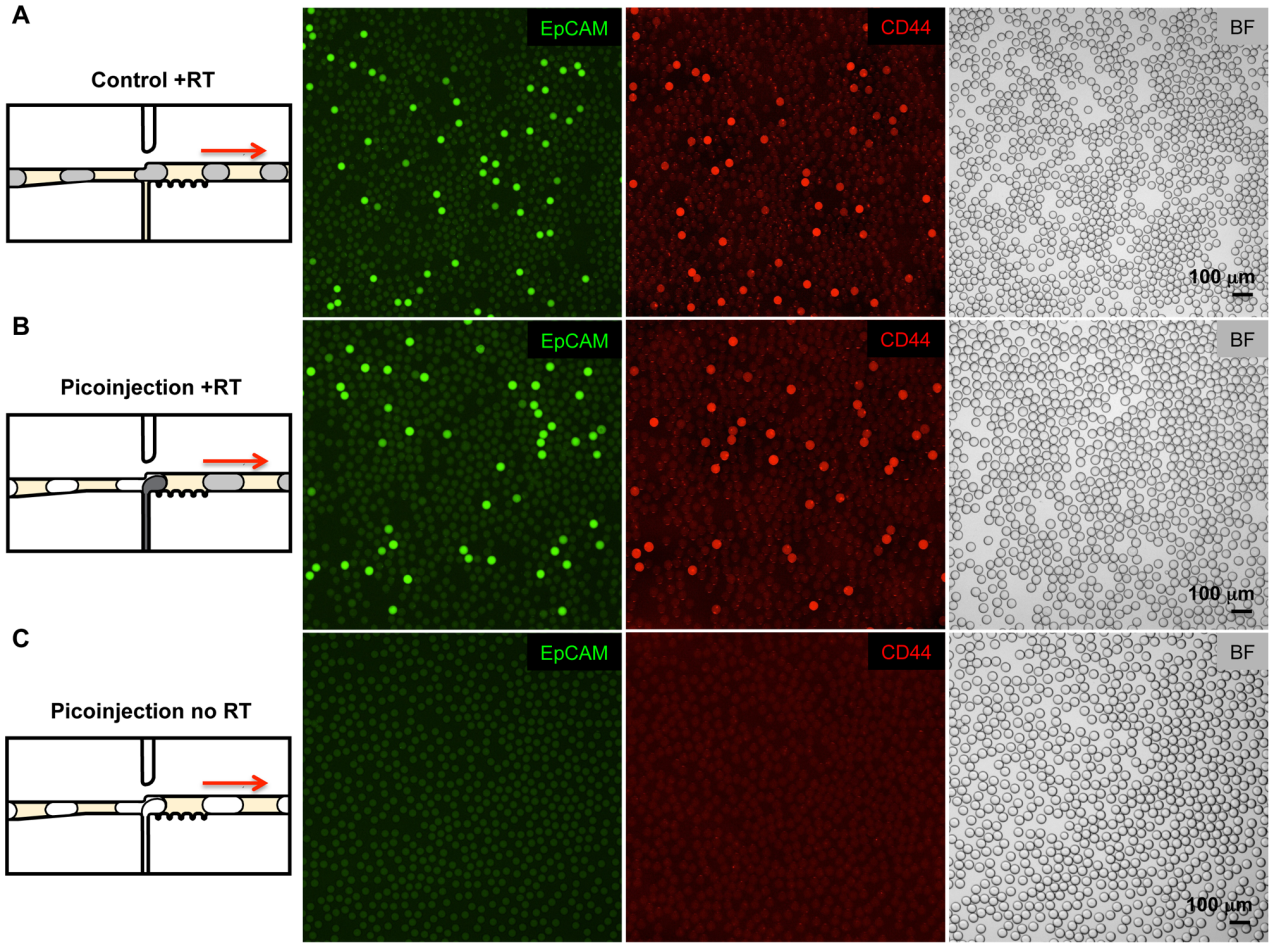
Digital RT-PCR Taqman assays in microfluidic drops following picoinjection of reverse transcriptase. (A) Control RT-PCR reactions containing PC3 cell total RNA were emulsified on a T-junction drop maker, thermocycled, and imaged. FAM (green) fluorescence indicates Taqman detection of an EpCAM transcript and Cy5 (red) indicates detection of CD44 transcripts. Brightfield images (BF) of the same drops are shown in the image panel on the far right. The red arrows indicate the direction of emulsion flow in the illustrations. (B) RT-PCR reactions lacking reverse transcriptase were emulsified on a T-junction drop maker and subsequently picoinjected with reverse transcriptase. Picoinjection fluid is pictured as dark gray in the schematic diagram on the left. Brightfield images demonstrate that the drops roughly doubled in size after picoinjection. (C) RT-PCR reactions subjected to picoinjection omitting the reverse transcriptase show no Taqman signal for EpCAM and CD44, demonstrating the specificity of the Taqman assay. Scale bars  = 100 µm.

To prepare the non-picoinjected control drops, we added the probe mix to a 25 µL RT-PCR master mix reaction containing 150 ng of total RNA isolated from the human PC3 prostate cancer cell line. We then emulsified the RT-PCR solution into monodisperse 30 µm (14 pL) drops with a T-junction drop maker [12], [13], and the drops were collected into PCR tubes and thermocycled (Figs. 1A and 1C). During thermocycling, drops containing at least one EpCAM or CD44 transcript were amplified, becoming fluorescent at the wavelengths of the associated FAM and Cy5 dyes. By contrast, drops without a molecule do not undergo amplification and remained dim, as in standard Taqman-based digital droplet RT-PCR [14]–[16]. Following thermocycling, the drops were pipetted into chambered slides and imaged with a fluorescence microscope. To measure the concentrations of EpCAM and CD44 in the original solution, we counted the number of drops with FAM or Cy5 fluorescence. The reactions showed a digital fluorescent signal for both the EpCAM and CD44 probes, indicating that these transcripts were present at limiting concentrations in the drops, as shown in Fig. 2A. Additional droplet based RT-PCR experiments using limiting dilutions of *in vitro* transcribed RNA support the conclusion that our reaction conditions enable single molecule RNA detection (data not shown).

To test the impact of picoinjection on Taqman RT-PCR, we performed a similar experiment as above, but separated the RT-PCR reagents into two solutions added at different times. We first emulsified total RNA, RT-PCR buffer, primers, probes, and DNA polymerase into 30 µm diameter drops; these drops were not capable of RT-PCR, since they lacked reverse transcriptase. Using picoinjection, we introduced an equal volume of 2X reverse transcriptase in PCR buffer and thermocycled the drops. Just as with the non-picoinjected control, this emulsion showed a robust digital signal and had an equivalent ratio of fluorescent-to-non-fluorescent drops, as shown in Figs. 2A and 2B. To confirm that the fluorescence is not due to background hydrolysis of the Taqman probes, disruption of the probes by the electric field, or some other factor, we performed additional reactions where a picoinjection fluid lacking reverse transcriptase was added to RNA-containing drops. In these drops, no fluorescence was evident following thermocycling (Fig. 2C), demonstrating that the signal was indeed a result of digital detection of RNA molecules and that our assays were specific.

### Quantification of RT-PCR detection rates in picoinjected drops

**Figure 3:**
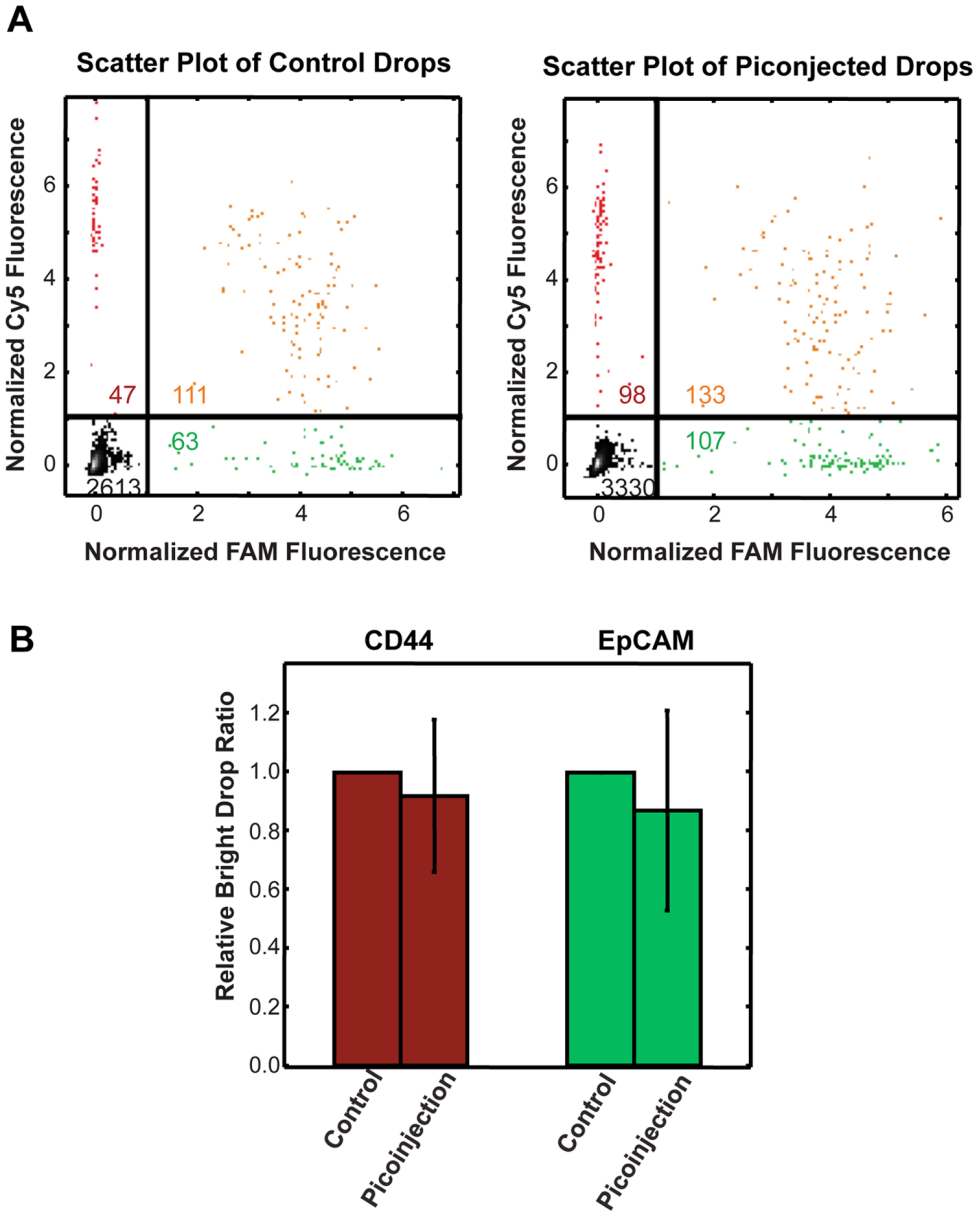
Comparison of digital RT-PCR detection rates between control drops and drops that were picoinjected with reverse transcriptase. (A) Scatter plots of FAM and Cy5 drop intensities for a control sample (left) and picoinjected sample (right). The gating thresholds used to label a drop as positive or negative for Taqman signal are demarcated by the lines, and divide the scatter plot into quadrants, double negative drops (–,–), FAM positive (–,+), Cy5 positive (+,–), positive for both FAM and Cy5 (+,+). Numbers of drops in each quadrant are indicated. (B) The bar graph shows the average Taqman positive drop count with picoinjection relative to the normalized count for CD44 and EpCAM Taqman assays for control populations. The control detection rate value is defined as 1 for each replicate. The data represent the average of four independent experimental replicates.

To precisely quantify the impact of picoinjection on Taqman RT-PCR transcript detection, we collected four independent replicates of the picoinjected and non-picoinjected drops. To automate data analysis, we used custom MATLAB software to locate the drops in the images and measure their fluorescence intensities. For a particular channel (FAM or Cy5), we averaged the fluorescence intensity within each drop; we then normalized all drop values so that the large cluster of Taqman negative drops had an average fluorescence intensity of zero (Materials and Methods). We then established a threshold fluorescence intensity for FAM and Cy5 channels. Drops were counted as positive or negative for EpCAM and CD44 fluorescence based on whether they were above or below the threshold, respectively, as shown in Fig. 3A. In total, we analyzed 16,216 control drops and 14,254 picoinjected drops from the four experimental replicates. To determine the Taqman detection rate of picoinjected drops relative to non-picoinjected controls, we first defined the total number of CD44 (Cy5) and EpCAM (FAM) positive drops in each of the control (non-picoinjected) replicates as 100% detection. Following picoinjection of reverse transcriptase, we detected 92% (+/−26%) of CD44 positive drops and 87% (+/−34%) of EpCAM positive drops relative to the control drops (Fig. 3B). Although the average transcript detection rate for picoinjected drops was slightly lower than that of control drops for a given RNA concentration, the difference was not statistically significant, and some experimental replicates had detection rates for picoinjected drops higher than for the controls. Based on our results, we conclude that picoinjection affords transcript detection rates equivalent to that of digital RT-PCR, with the benefit of allowing the reaction components to be added at different times.

### Discrete populations of drops can be picoinjected with minimal cross-contamination

**Figure 4:**
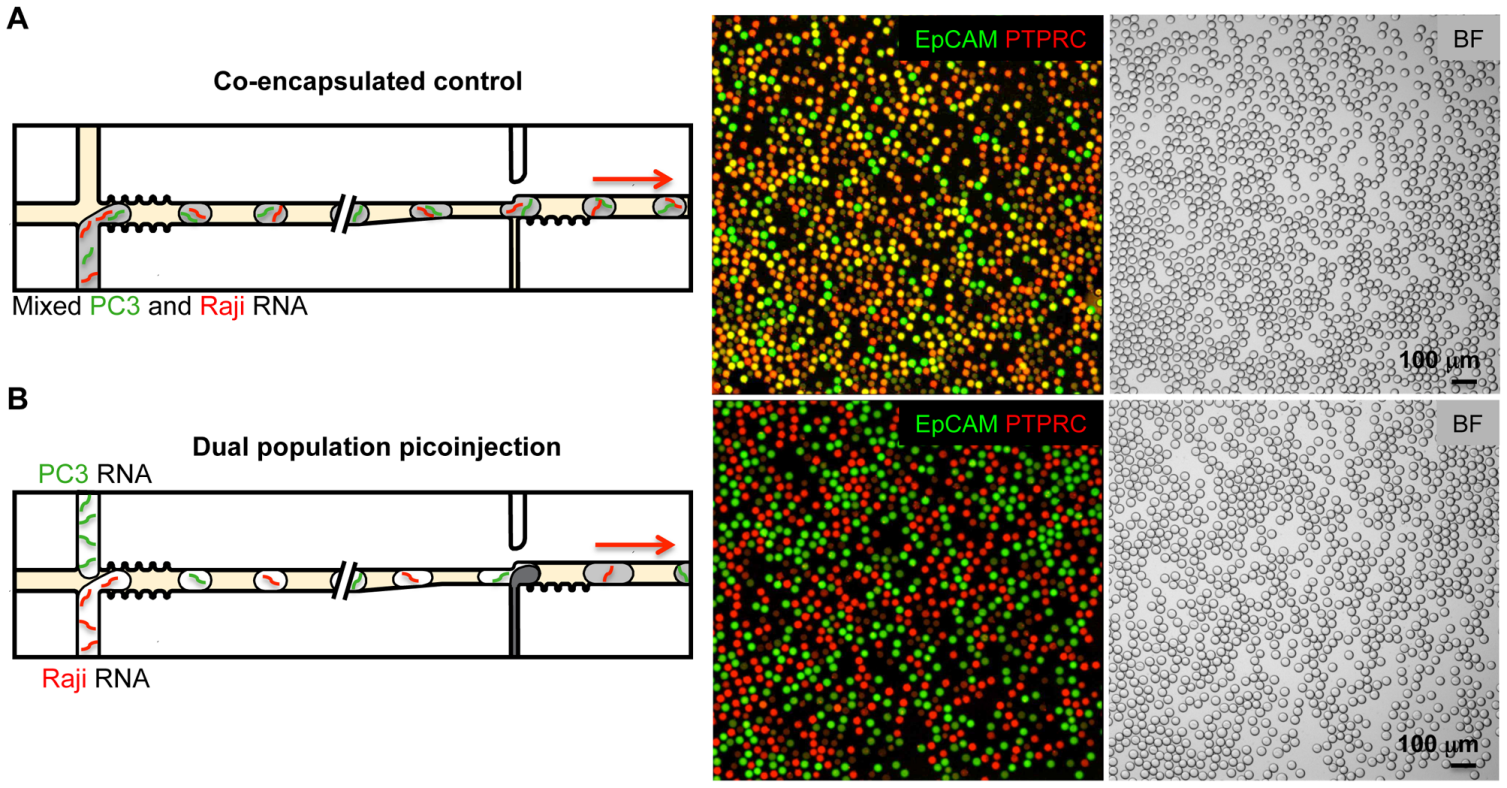
Picoinjection enables analysis of discrete drop populations. (A) Non-picoinjected drops. Control RT-PCR reactions containing mixed PC3 cell total RNA and Raji cell total RNA were emulsified with a T-junction drop maker, thermocycled, and imaged. Merged FAM and HEX fluorescent images are shown with FAM (green) fluorescence indicating Taqman detection of an EpCAM transcript and HEX (red) indicating the presence of PTPRC transcripts. The yellow drops indicate the presence of multiplexed Taqman assays, where EpCAM and PTPRC transcripts were co-encapsulated in the same drop. The brightfield images (BF) are shown in the panel on the right. The red arrows indicate the direction of emulsion flow in the illustrations. (B) Picoinjected drops. A double T-junction drop maker simultaneously created two populations of drops that were immediately picoinjected. One drop maker created drops containing only Raji cell RNA, and the other drops containing only PC3 cell RNA. Both drop types initially lack reverse transcriptase, which is added via picoinjection just downstream of the drop makers. The overwhelming majority of drops display no multiplexing, demonstrating that transfer of material during picoinjection is very rare. Scale bars  = 100 µm.

An important feature when adding reagents to drops is maintaining the unique contents of each drop and preventing the transfer of material between drops. Unlike the merger of two discrete drops, the contents of a picoinjected drop become momentarily connected with the fluid being added, as illustrated in Fig. 1B. After the drop disconnects from the fluid, it may leave material behind that, in turn, may be added to the drops that follow. This could lead to transfer of material between drops, and cross-contamination. To examine the extent to which picoinjection results in cross-contamination, we again used Taqman RT-PCR reactions because they are extremely sensitive and capable of detecting the transfer of just a single RNA molecule. We used a FAM-conjugated Taqman probe targeting the EpCAM transcript and a hexachlorofluorescein (HEX) conjugated Taqman probe recognizing the B-lymphocyte-specific transcript PTPRC. We isolated total RNA from PC3 cells expressing EpCAM but not PTPRC, and a B-lymphocyte derived cell line (Raji) expressing PTPRC but not EpCAM. For a control set of drops, we mixed the RNA from both cell types, added the Taqman probes and RT-PCR reagents, and emulsified the solutions into 30 µm drops. The drops were collected into a tube, thermocycled, and imaged, Fig. 4A. In the images, a large number of drops displayed FAM and HEX fluorescence, indicative of multiplexed Taqman detection of PTPRC and EpCAM transcripts. A smaller fraction had pure green or red fluorescence, indicating that they originally contained just one of these molecules, while even fewer were dim and were thus devoid of these transcripts.

To observe the rate of picoinjector cross-contamination, we used a microfluidic device that synchronously produced two populations of drops from opposing T-junctions [17], pictured in Fig. 1A and 4B. One population contained only Raji cell RNA and PTPRC transcripts; the other, only PC3 cell RNA and EpCAM transcripts, as illustrated in Fig. 4B. Both populations contained primers and Taqman probes for EpCAM and PTPRC and were therefore capable of signaling the presence of either transcript. Immediately after formation, the drops were picoinjected with the 2X reverse transcriptase, thereby enabling first strand cDNA template synthesis for the Taqman assay, and an opportunity for contamination. If RNA was transferred between drops, some of the drops should display a multiplexed Taqman signal, whereas in the absence of contamination, there should be two distinct populations and no multiplexing. In the fluorescence images, we saw two distinct populations, one positive for EpCAM (FAM) and the other for PTPRC (HEX), with almost no yellow multiplexed drops that would be indicative of a multiplexed signal, as shown in Fig. 4B. This demonstrates that cross-contamination during picoinjection is rare.

**Figure 5:**
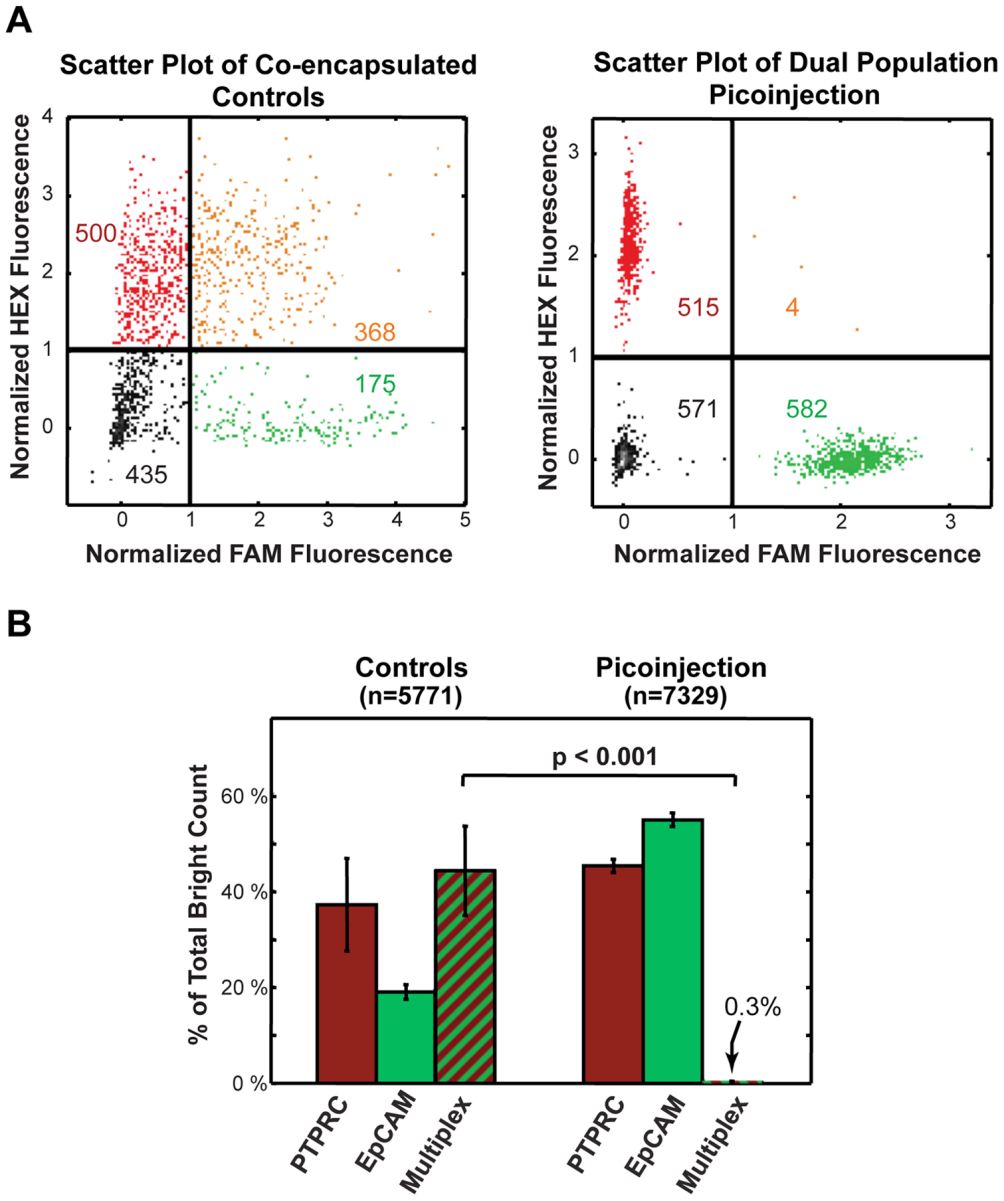
Dual transcript detection analysis indicates minimal cross-contamination during picoinjection. (A) Scatter plots of FAM and HEX drop intensities for a co-encapsulated control sample (left) and dual population picoinjected sample (right). Using this analysis, large numbers of Taqman multiplexed drops were identified in the co-encapsulated controls that were virtually absent in the dual population picoinjected drops (upper right quadrants of gated scatter plots). (B) A bar graph of different bright drop populations relative to the total bright count for co-encapsulation control and for dual population picoinjection. The data represent the average of three experimental replicates.

To measure the precise rate of cross-contamination, we used our automated droplet detection software to analyze thousands of drops, Fig. 5A, and plotted the results as a percentage of the total number of Taqman positive drops, Fig. 5B. We analyzed a total of 5,771 Taqman positive control drops and 7,329 Taqman positive picoinjected drops from three independent experimental replicates. For the control drops, in which we combined the Raji and PC3 RNA, we observed a multiplexing rate 44.1% (+/−9.3). By contrast, for the picoinjected drops, we observed only 0.3% (+/−0.1) multiplexed drops, as shown in Fig. 5B. Hence, with picoinjection, there is some multiplexing, although the rate is so low we cannot rule out other sources of RNA transfer, such as merger of drops during thermocycling or transport of RNA between droplet interfaces.

**Figure 6:**
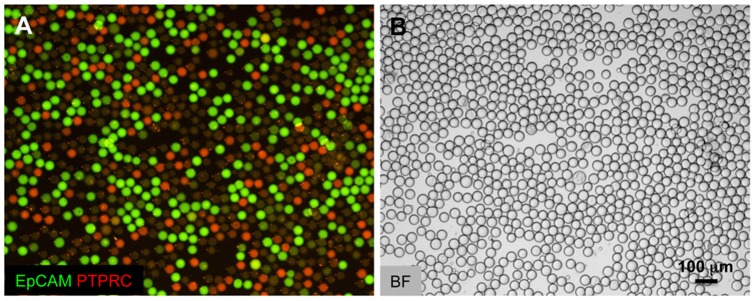
Dual populations of RNA drops can be stored offline and picoinjected at a later time. (A) An emulsion was made consisting of two populations of drops, one containing RNA recovered from Raji cells, and the other from PC3 cells. The drops were collected into a syringe, incubated off chip, and then re-introduced into a microfluidic device to picoinject. The drops were then collected, thermocycled, and imaged. These drops are somewhat more polydisperse and displayed higher multiplexing rates (1%) than the drops picoinjected on the same device on which they were formed, which is most likely due to merger of some of the drops during incubation and reinjection. The ability to reinject emulsions following incubation to add reagents is critical for numerous droplet-based molecular biology assays. (B) Brightfield images of picoinjected emulsions. Scale bars  = 100 µm.

Our dual population experiments in which the drops were picoinjected immediately after being formed allowed us to estimate the precise amount of cross-contamination, but in most actual implementations of picoinjection for biological assays, the drops will be formed on one device, removed offline for incubation or thermocycling, and then reinjected into another device for picoinjection. To demonstrate that picoinjection is effective for digital RT-PCR reactions performed under these conditions, and to estimate the rate of cross-contamination, we again created a dual population of drops, but this time pulled the drops offline and stored them in a 1 mL syringe before reinjecting and picoinjecting them. Just as before, we observed that nearly all drops were pure green or red, indicating minimal cross-contamination, as shown in Fig. 6. However, we also found some drops with a multiplexed signal, as shown by the rare yellow drops in the image. In this experiment, the multiplexing rate was 1%, higher than with the drops that were picoinjected immediately after formation. While we cannot rule out cross-contamination at the picoinjector, we suspect the higher multiplexing rate to be the result of merger of drops during offline storage and reinjection, during which the drops are subjected to dust, air, and shear forces that can increase the chances for merger. This is supported by our observation that during reinjection of the emulsion there were occasional large merged drops, and also that the picoinjected emulsion was somewhat polydisperse, as shown in Fig. 6. Nevertheless, even under these rough conditions, the vast majority of drops displayed no multiplexing, indicating that they retained their integrity as distinct reactors.

### Conclusion

We have demonstrated that picoinjection is compatible with droplet digital RT-PCR and affords single RNA molecule detection rates equivalent to workflows not incorporating picoinjection. This shows that picoinjection is compatible with reactions involving common biological components, like nucleic acids, enzymes, buffers, and dyes. We also found that there is negligible transfer of material between drops during picoinjection. Our results support picoinjection as a powerful and robust technique for adding reagents to drops for ultrahigh-throughput biological assays. Furthermore, workflows where populations of cells are first encapsulated in drops, lysed and subsequently picoinjected with PCR reagents could enable rare cell detection from heterogeneous populations of cells.

## Materials and Methods

### Microfluidic device fabrication

The microfluidic devices consisted of polydimethylsiloxane (PDMS) channels bonded to a glass slide [18]. To make the PDMS mold, we first created a device master by spinning a 30 µm-thick layer of photoresist (SU-8 3025) onto a silicon wafer, followed by a patterned UV exposure and resist development. We next poured an uncured mix of polymer and crosslinker (10:1) over the master and baked at 80°C for 1 hour. After peeling off the cured mold, we punched access holes in the PDMS slab with a 0.75 mm biopsy coring needle. We washed the device with isopropanol, dried it with air, and then bonded it to a glass slide following a 20 s treatment of 1 mbar O_2_ plasma in a 300 W plasma cleaner. To make the devices hydrophobic, we flushed the channels with Aquapel and baked them at 80°C for 10 min.

### RNA isolation

Human PC3 prostate cancer or Raji B-lymphocyte cell lines were cultured in appropriate growth medium supplemented with 10% FBS, penicillin and streptomycin at 37°C with 5% CO_2_ [19], [20]. Prior to RNA isolation, Raji cells were pelleted and washed once in phosphate buffered saline (PBS). Confluent and adhered PC3 cells were first trypsinized prior to pelleting and washing. Total RNA was isolated from cell pellets using an RNeasy Mini Kit (Qiagen). Total RNA was quantified using a spectrophotometer and the indicated amounts (between 150 and 1000 ng) of RNA were used in subsequent 25 µL RT-PCR reactions.

### Taqman RT-PCR reactions

The sequence of amplification primers used for the RT-PCR reactions were as follows: EpCAM Forward 5′-CCTATGCATCTCACCCATCTC-3′, EpCAM Reverse 5′-AGTTGTTGCTGGAATTGTTGTG-3′; CD44 Forward 5′-ACGGTTAACAATAGTTATGGTAATTGG-3′, CD44 Reverse 5′-CAACACCTCCCAGTATGACAC-3′; PTPRC/CD45 Forward 5′-CCATATGTTTGCTTTCCTTCTCC-3′, PTPRC/CD45 Reverse 5′-TGGTGACTTTTGGCAGATGA-3′. All PCR primers were validated prior to use in microfluidic droplet experiments with tube-based RT-PCR reactions. Products from these reactions were run on agarose gels and single bands of the predicted amplicon size were observed for each primer set. The sequence of Taqman probes are as follows: EpCAM 5′-/6-FAM/ATCTCAGCC/ZEN/TTCTCATACTTTGCCATTCTC/IABkFQ/-3′; CD44 5′-/Cy5/TGCTTCAATGCTTCAGCTCCACCT/IAbRQSp/-3′; PTPRC/CD45 5′-/HEX/CCTGGTCTC/ZEN/CATGTTTCAGTTCTGTCA/IABkFQ/-3′. Pre-mixed amplification primers and Taqman probes were ordered as a PrimeTime Standard qPCR assay from Integrated DNA Technologies (IDT) and were used at the suggested 1X working concentration. Superscript III reverse transcriptase (Invitrogen) was added directly to PCR reactions to enable first stand cDNA synthesis. Following emulsification or picoinjection of RT-PCR reagents, drops were collected in PCR tubes and transferred to a T100 Thermal Cycler (BioRad). Reactions were incubated at 50°C for 15 min followed by 93°C for 2 min and 41 cycles of: 92°C, 15 s and 60°C, 1 min. To prevent evaporation of PCR reactions from the microfluidic drops, we used the heated lid on the thermocycler set to 105°C. With the heated lid on, we were unable to detect any evaporation or drop shrinkage.

### Emulsion generation and picoinjection

The reaction mixtures were loaded into 1 mL syringes and injected into microfluidic T junction drop makers using syringe pumps (New Era) controlled with custom LabVIEW software. The dimensions of the device and flow rates of the reagents were adjusted to obtain the desired 30 µm drop size. To apply the electric field for picoinjection, we filled the electrode and surrounding moat channels with a 3 M NaCl solution, having a conductivity of ∼0.1 S/cm. We energized the electrode using 20 kHz, 300 VAC signals generated by a fluorescent light inverter (JKL Components Corp) attached via an alligator clip to the syringe needle.

### Immunofluorescence imaging

To image the thermocycled droplets, 10 µL of emulsion were pipetted into Countess chambered coverglass slides (Invitrogen). The slides were imaged on a Nikon Eclipse Ti inverted microscope using conventional widefield epifluorescence and a 4x objective. Fluorescence filters were chosen to optimize the signal intensity and to mitigate background fluorescence due to spectral overlapping of the dyes used in the multiplexed reactions. The images were captured using NIS Elements imaging software from Nikon.

### Data analysis

The droplet images were analyzed using custom MATLAB software. For each field of view, brightfield and fluorescence images were captured. The software first located all drops in the brightfield image by fitting circles to the drop interfaces. Next, the light background in the fluorescence images was subtracted using a smooth polynomial surface constrained to vary over size scales much larger than the drops. The software then measured the average fluorescence intensity within each droplet's circular boundary. The resultant intensity values were offset so that the cluster of lowest intensity (empty) had an average of zero. Drops were determined to be “positive” or “negative” based on whether their intensity fell above or below, respectively, a defined threshold.
